# 

**Published:** 1878-02-20

**Authors:** 


☞All communications relating to the business of The Record, for the years 1877 and 1878, must be addressed to DR. R. C. WORD, Managing Editor Southern Medical Record, Atlanta, Ga.

☞Brief and practical communications are solicited on all subjects pertaining to medicine; also reports of cases in practice.

☞Send money by check, postal order or registered letter.

☞Write your name, post-office, county and State plainly.

### RECEIPTED.

N J Long, H Perdue, A W Mann, I H Goss, J C Wallace, John Riches, Jno Vandergriff, S W Eaton, J S Bacon, Joseph Underwood, W J Lee, L K Branch, J B Foster, S B Wall, D R Fox, W W Ward, H Allison, I H Wysong, G F Taylor, Preston Pond, J I Groover, John Slaughter, Lewis Hadden, J Dellworth, C F Rogers, M L Barron, T S Roach, B M Bledsoe, J W Unger, R H Lee, W T Kendall, W H Russell, W B Williamson, R E Hutchins, McCabe & Lanier, J E G Terrell, B R Doyle, J H Calfee, W H Calfee, T J Jones, W P Lawton, J R Johnson, S Y T Carter, W T Mathes, E H Hale, A H Sellers.

